# Systematic Review of Cost-Effectiveness Models in Prostate Cancer: Exploring New Developments in Testing and Diagnosis

**DOI:** 10.1016/j.jval.2021.07.002

**Published:** 2022-01

**Authors:** Edna Keeney, Howard Thom, Emma Turner, Richard M. Martin, Josie Morley, Sabina Sanghera

**Affiliations:** 1 Health Economics Bristol, Bristol Medical School, University of Bristol, Bristol, England, UK; 2 Population Health Sciences, Bristol Medical School, University of Bristol, Bristol, England, UK; 3 MRC Integrative Epidemiology Unit, Bristol Medical School, University of Bristol, Bristol, England, UK

**Keywords:** cost-effectiveness models, diagnosis, prostate cancer, systematic review

## Abstract

**Objectives:**

Recent innovations in prostate cancer diagnosis include new biomarkers and more accurate biopsy methods. This study assesses the evidence base on cost-effectiveness of these developments (eg, Prostate Health Index and magnetic resonance imaging [MRI]-guided biopsy) and identifies areas of improvement for future cost-effectiveness models.

**Methods:**

A systematic review using the National Health Service Economic Evaluation Database, MEDLINE, Embase, Health Technology Assessment databases, National Institute for Health and Care Excellence guidelines, and United Kingdom National Screening Committee guidance was performed, between 2009 and 2021. Relevant data were extracted on study type, model inputs, modeling methods and cost-effectiveness conclusions, and results narratively synthesized.

**Results:**

A total of 22 model-based economic evaluations were included. A total of 11 compared the cost-effectiveness of new biomarkers to prostate-specific antigen testing alone and all found biomarkers to be cost saving. A total of 8 compared MRI-guided biopsy methods to transrectal ultrasound-guided methods and found MRI-guided methods to be most cost-effective. Newer detection methods showed a reduction in unnecessary biopsies and overtreatment. The most cost-effective follow-up strategy in men with a negative initial biopsy was uncertain. Many studies did not model for stage or grade of cancer, cancer progression, or the entire testing and treatment pathway. Few fully accounted for uncertainty.

**Conclusions:**

This review brings together the cost-effectiveness literature for novel diagnostic methods in prostate cancer, showing that most studies have found new methods to be more cost-effective than standard of care. Several limitations of the models were identified, however, limiting the reliability of the results. Areas for further development include accurately modeling the impact of early diagnostic tests on long-term outcomes of prostate cancer and fully accounting for uncertainty.

## Introduction

Prostate cancer is the second most commonly occurring cancer in men worldwide and the fourth most commonly occurring cancer overall.^1^ Detection of early disease has historically been achieved using a prostate-specific antigen (PSA) blood test followed by transrectal ultrasound (TRUS)-guided biopsy. Nevertheless, PSA is not a specific marker for prostate cancer, and TRUS-guided prostate biopsy is associated with infection and other adverse effects and can lead to false negative results in up to 25% of cases.^2^^,^^3^ Therefore, current diagnostic methods lead to overdetection of cancers that may not progress to become clinically important in a man’s lifetime, but can also miss aggressive, potentially fatal prostate cancer.^4^^,^^5^ Overdetection can have a significant effect on the quality of life (QOL) of the men affected owing to the adverse effects associated with testing and unnecessary treatment.^6^ It is also a poor use of limited healthcare resources. In the absence of robust evidence, current UK policy does not advocate population screening. Several large trials, including the European Randomised Study of Screening for Prostate Cancer (ERSPC),^7^ the US Prostate, Lung, Colorectal, and Ovarian Cancer Screening Trial,^8^ and the UK Cluster Randomized Trial of PSA Testing for Prostate Cancer trial,^5^ have found limited mortality benefit of PSA-based screening when considered overall.^9^ Therefore, as it stands, the benefits of screening seem insufficient to outweigh the potential harms of overtreatment.

Recent years have seen the development of biomarker tests to complement PSA-based testing, for example, the Prostate Health Index (PHI), 4Kscore, SelectMDx, and PCA3. These tests act as additional reflex tests to aid the decision about when a man should be referred for prostate biopsy. Multiparametric magnetic resonance imaging (MRI) is another recent development that, when used as a triage test after PSA or other biomarker testing, might allow men with no or likely indolent cancer to avoid unnecessary biopsy and improve diagnostic accuracy with respect to more aggressive disease.^10^^,^^11^ Therefore, there is potential for a reduction in overdiagnosis and higher specificity for potentially lethal cancer.^4^ Nevertheless, it is not yet clear whether these new developments should be implemented either individually or in combination with one another at a national level within a screening program.

As innovations that aim to address the overdiagnosis associated with prostate cancer screening become available, healthcare policy makers must make informed decisions regarding their use in national screening strategies. As such, it is essential to establish the cost-effectiveness of these developments and their combinations, to make rational decisions about the allocation of limited healthcare resources.

This systematic review aimed to identify published economic models assessing the impact of these innovations on the costs and outcomes of prostate cancer diagnosis. The population of interest was men at risk of developing prostate cancer, the interventions reviewed were novel biomarkers and MRI-guided biopsy techniques as prostate cancer diagnostic tools, the alternatives against which the interventions were compared were standard diagnostic tools such as the PSA test, TRUS-guided biopsy, or no intervention, and the outcome considered was the cost-effectiveness of these interventions in comparison with each other. This review also determines the current evidence base and provides an overview of model characteristics. It provides information on novel tests, how they have been modeled, and the data available to populate such models, which will assist the development of new cost-effectiveness models in prostate cancer screening. It assesses the limitations of available models, highlighting ways in which a future model may improve on these, and provides overall conclusions on the cost-effectiveness of these new diagnostic tools.

## Methods

### Study Selection

Study selection proceeded from title/abstract screening against the eligibility criteria through full-text review to data extraction. E.K. was involved at all stages. A second reviewer (J.M.) independently screened 10% of the titles and abstracts and performed data extraction on 20% of the included studies. Studies were categorized according to model-based economic evaluations of new (1) biomarkers/tests/risk models for screening in prostate cancer, (2) biopsy methods for definitive diagnosis after an initial triage screening test in prostate cancer, and (3) follow-up testing and diagnostic strategies for men initially found to have no or low-risk prostate cancer.

### Search Strategy

In April 2021, studies were identified by searching the National Health Service Economic Evaluation Database (2009-2014), MEDLINE, Embase, Health Technology Assessment databases, National Institute for Health and Care Excellence (NICE) guidelines, UK National Screening Committee guidance, and reference lists from relevant studies. The review was restricted to evidence from January 2009 onward to reflect current practice in screening and testing for prostate cancer and because the aim was to identify novel tests in prostate cancer diagnosis. Search terms included free text and medical subject headings terms (Appendix 1 in Supplemental Materials found at https://doi.org/10.1016/j.jval.2021.07.002). The search was limited to English language publications.

### Eligibility Criteria

Studies were included if they were model-based economic evaluations of screening or diagnostic strategies for prostate cancer beyond the standard PSA test plus TRUS-guided biopsy. Cost-effectiveness, cost-utility, cost-consequence, and cost-benefit analyses were considered. Models could use primary data from a trial or secondary data from the literature. They could compare any novel test or diagnostic strategy for diagnosing or ruling out prostate cancer or any subsequent follow-up regime (aside from PSA testing) when prostate cancer has not been identified at initial biopsy. Models from any country or type of health system were considered.

### Data Extraction

Data extraction forms were developed and pilot-tested on a random sample (5%) of included studies and refined accordingly. The data extraction form is shown in Appendix 2 in Supplemental Materials found at https://doi.org/10.1016/j.jval.2021.07.002.

Information extracted from each study included context (ie, perspective and country), characteristics of the tests compared (ie, frequency of testing and threshold for a positive result), the population the strategy was applied to (ie, screening start and stop age and the prevalence of prostate cancer), outcome measures (eg, cost per quality-adjusted life-year [QALY] gained), and cost-effectiveness result. Information was also extracted on characteristics of the model including model type (eg, decision tree, Markov model) and structure (how clinical pathways are represented), sensitivity analyses (including the extent to which uncertainty in the cost-effectiveness result had been quantified), the source of evidence for utility values assigned to health states and costs included in the model, and the source of evidence for accuracy of tests.

### Quality Assessment

The purpose of the review was to determine the current evidence base and provide an overview of the characteristics of available models. Therefore, a formal quality checklist was not used to exclude studies from the review. Nevertheless, existing economic evaluation checklists were used as a guide to reporting the studies.^12^^,^^13^ The quality of the included economic evaluations was assessed using the Consolidated Health Economic Evaluation Reporting Standards checklist.^14^ A score of 0, 1, or 2 was allocated for each criterion corresponding to a decision of criterion not met, criterion met, or criterion not applicable. Risk of bias was assessed using the Bias in Economic Evaluation checklist.^15^ Every item was rated as yes, no, partly, unclear, or not applicable.

The review follows the reporting standards for reviews of economic evaluations.^16^, ^17^, ^18^

## Results

**Figure 1:**
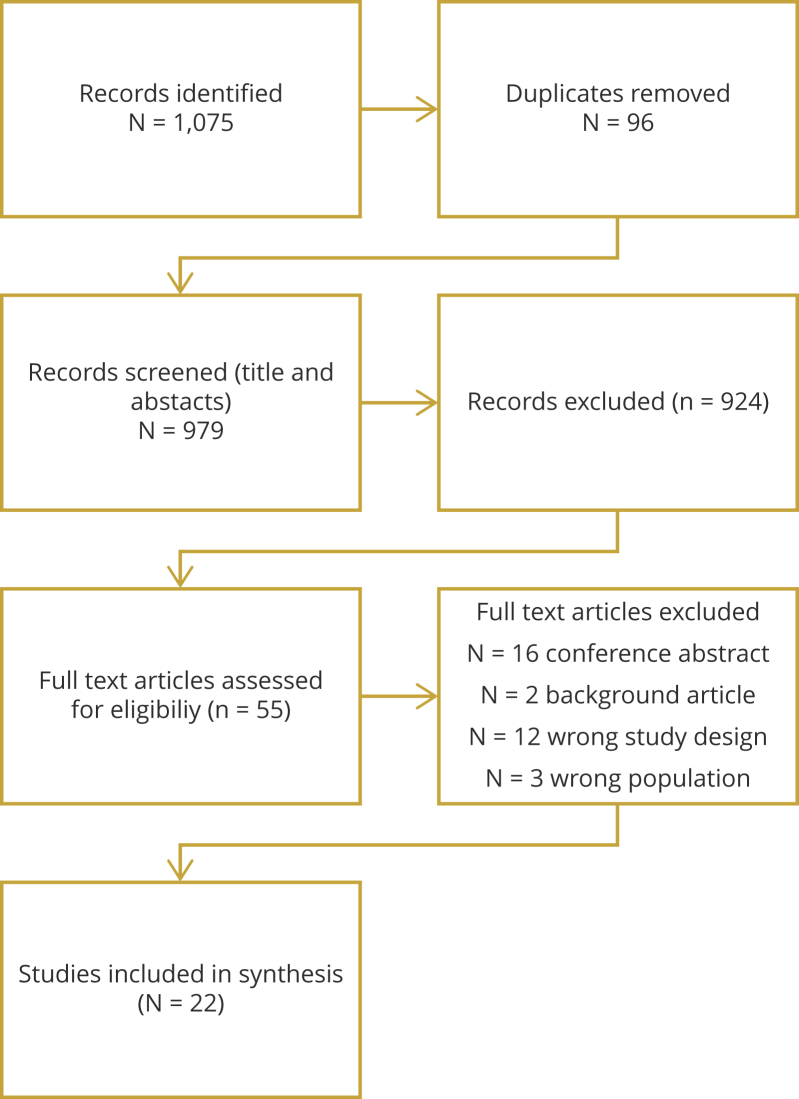
Studies included and excluded from the review.

In total, 1075 studies were identified. Most studies were excluded at the abstract stage because they were not model-based economic evaluations or did not compare tests for diagnosing prostate cancer. After removing duplicates and checking for eligibility, 55 full-text articles were retrieved (Fig. 1). Of the 55 full-text articles, 22 studies were included in the review. A total of 16 articles were excluded because these were conference abstracts and the rest were excluded because they (1) were background articles rather than original studies, (2) had the wrong study design, for example, cost-comparison rather than cost-effectiveness analyses, or (3) had the wrong population, for example, men with biochemical recurrence after radical prostatectomy.

### Study Type

Of the 22 studies, 11 compared the cost-effectiveness of new urinary or blood biomarkers to each other or to the standard of care (a PSA test alone) (Table 1). Another 8 studies compared different approaches with prostate biopsy. A total of 3 studies compared follow-up strategies in men who have a negative initial biopsy result. The studies were based in the United States (n = 6), United Kingdom (n = 6), The Netherlands (n = 4), Hong Kong (n = 1), Germany (n = 1), China (n = 1), Sweden (n = 1), and Canada (n = 1). One study compared results for France, Germany, Spain, and Italy.^19^ All but 3 studies^20^, ^21^, ^22^ performed a cost-utility analysis where outcomes were measured in QALYs. The other 3 were cost-consequence analyses reporting the number of tests and biopsies performed and expected overall diagnostic costs.^20^^,^^21^

**Table 1:** Characteristics of studies included after full-text screening

Author	Year	Country	Patient population	Age	Assumed prevalence of PCa, %	Strategies compared
Biomarkers						
Bouttell et al^20^	2019	Hong Kong	Normal DRE, PSA 4-10 ng/mL	NR	10.9	1. Biopsy all 2. Biopsy only if PHI >25 3. Biopsy only if PHI >35 4. Biopsy only if PHI >55
Govers et al^26^	2018	US	Elevated PSA or abnormal DRE	NR	46.4	1. Biopsy all 2. Biopsy only if SelectMDx +
Sathianathen al^28^	2018	US	PSA >3 ng/mL	50	29	1. Biopsy all 2. Biopsy only if SelectMDx + 3. Biopsy only if PHI + 4. Biopsy only if EPI + 5. Biopsy only if 4Kscore +
Dijkstra et al^25^	2017	Holland	PSA >3 ng/mL	NR	44.4	1. Biopsy all 2. Biopsy only if SelectMDx +
Heijnsdijk et al^23^	2016	Holland	PSA >3 ng/mL	50-75	NR	1. Biopsy all 2. Biopsy only if PHI >25
Schiffer et al^21^	2012	Germany	PSA >4 and/or suspicious DRE in a urological outpatient center setting	66	24	1. Biopsy all 2. Biopsy only if UPA-PC +
Nichol et al^24^	2011	US	PSA 2-10 ng/mL	50-75	25	1. Biopsy all 2. Biopsy only if PHI +
			PSA 4-10 ng/mL	50-75	25	
			PHI+ at PSA 2-10 ng/mL	50-75	29.6	
			PHI+ at PSA 4-10 ng/mL	50-75	30.3	
			PSA >10 ng/mL	50-75	66.70	
Kim et al^22^	2020	UK	Referred from primary care for elevated PSA	66	NR	1. mpMRI and biopsy all 2. mpMRI all and biopsy if positive 3. mpMRI all and biopsy if PSA density ≥0.15 4. mpMRI all and biopsy if PSA density ≥0.1 5. PHI all and mpMRI and biopsy if PHI ≥25 6. PHI all and mpMRI and biopsy if PHI ≥30
Teoh et al^39^	2020	China	Patients with normal DRE undergoing opportunistic PSA testing	50-75	NR	1. Biopsy if PSA 4-10 ng/mL 2. Biopsy only if PSA 4-10 ng/mL and PHI >35
Karlsson et al^27^	2021	Sweden	All men	55-69	NR	1. No screening 2. Quadrennial screening for men at the age of 55-69 years with PSA test alone 3. Quadrennial screening for men at the age of 55-69 years with PSA test and reflex Stockholm3 test for PSA values above 1, 1.5, and 2 ng/mL, respectively
Govers et al^19^	2019	France, Germany, Italy, and Spain	Men who, under current guideline concordant management, would undergo initial TRUS-guided biopsy	NR	France: 47 Germany: 49 Italy: 37 Spain: 33	1. Biopsy all 2. Biopsy only if SelectMDx +
Biopsy methods						
Barnett et al^32^	2018	US	Biopsy-naive men with PSA >4 ng/mL	55-69	NR	1. Standard biopsy for all 2. MRI, if positive targeted fusion biopsy 3. MRI, if positive combined biopsy For 2 and 3 additional strategies of no further biopsy or additional standard biopsy if negative
Pahwa et al^42^	2017	US	Biopsy-naive men recommended for prostate biopsy on basis of abnormal DRE or elevated PSA	41-50	37	1. Standard biopsy for all 2. MRI + cognitively guided biopsy 3. MR imaging/US fusion biopsy 4. in-gantry MR imaging-guided biopsy For 2-4 additional strategies of no further biopsy or additional standard biopsy if negative
				51-60	44	
				41-70	50	
				61-70	65	
Venderink et al^46^	2017	Holland	Biopsy-naive men with elevated PSA or abnormal DRE	NR	25	1. TRUS-guided biopsy for all 2. mpMRI, if suspicious MRI TRUS fusion-guided biopsy 3. Direct in-bore MRI-guided biopsy
Cerantola et al^38^	2016	Canada	Biopsy-naive men with clinical suspicion of PCa (based on DRE and PSA values 4-10 ng/mL) with life expectancy of 20 years	60-65	24	1. TRUS-guided biopsy for all 2. MRI-targeted biopsy
de Rooij et al^41^	2013	Holland	elevated PSA level ( > 4 ng/mL)	60	25	1. TRUS-guided biopsy for all 2. MRI-guided biopsy
Faria et al^30^	2018	UK	Men at risk of PCa referred to secondary care for further investigation	NR	38	383 clinically feasible combinations of mpMRI, TRUS-guided biopsy, and TPMB, in addition to the use of TRUS-guided biopsy and TPMB in isolation
Barnett et al^31^	2019	US	Biopsy-naive men with elevated PSA levels ( >4 ng/mL)	55-69	NR	1. Standard biopsy for all 2. mpMRI, if positive combined biopsy 3. Hybrid ^18^F-choline PET/mpMRI, if positive combined biopsy For 2 and 3 additional strategies of using Likert or PI-RADSv2 scores to determine positive results and no further biopsy or additional standard biopsy if negative
Callender et al^33^	2021	UK	All men	55-69	NR	1. No screening 2. Age-based screening with biopsy if PSA ≥3 3. Age-based screening with MRI if PSA ≥3 and biopsy if abnormal findings 4. Risk-stratified screening with biopsy if PSA ≥3 5. Risk-stratified screening with MRI if PSA ≥3 and biopsy if abnormal findings
Follow-up strategies in men with negative biopsies						
NICE Guideline^35^	2019	UK	Raised PSA, negative MRI, and/or negative prostate biopsy	66-75	58.2	Different follow-up strategies, including screening test (PSA density, velocity, doubling time, % free forms) PCA3 or PHI, at different frequencies and different thresholds for triggering further investigation; diagnostic stage possibly including MRI techniques
Nicholson et al^36^	2015	UK	Men referred for second biopsy because, after negative initial biopsy result, clinicians still suspect malignant PCa present	NR	24	1. clinical assessment 2. clinical assessment + PCA3 3. clinical assessment + PHI 4. clinical assessment + PCA3 + PHI 5. clinical assessment + mpMRI 6. clinical assessment + mpMRI + PCA3 7. clinical assessment + mpMRI + PHI 8. clinical assessment + mpMRI + PCA3 + PHI
Mowatt et al^37^	2013	UK	Suspected PCa with a prior negative/inconclusive biopsy, with indications for repeat biopsy (ie, sustained suspicion of PCa as a result of clinical and/or pathological findings)	60	24	1. TRUS-guided biopsy for all 2. T2-MRI 3. MRS 4. DCE-MRI 5. T2-MRI or MRS 6. T2-MRI or DCE-MRI

DCE-MRI indicates dynamic contrast-enhanced magnetic resonance imaging; DRE, digital rectal examination; EPI, ExoDx® Prostate(IntelliScore); mpMRI, multiparametric magnetic resonance imaging; MR, magnetic resonance; MRI, magnetic resonance imaging; MRS, magnetic resonance spectroscopy; NR, not reported; PCa, prostate cancer; PET, positron emission tomography; PHI, Prostate Health Index; PI-RADSv2, Prostate Imaging-Reporting and Data System version 2; PSA, prostate-specific antigen; TPMB, transperineal mapping biopsy; TRUS, transrectal ultrasound; UPA-PC, urinary proteome analysis for prostate cancer diagnosis; UK, United Kingdom; US, United States.

#### Strategies compared—biomarkers

The novel diagnostic strategies that were compared with PSA-based testing alone included PHI,^20^^,^^22^, ^23^, ^24^ PCA3, SelectMDx,^25^^,^^26^ Stockholm3,^27^ and urinary proteome analysis.^21^ The definitions of these biomarker tests are given in Appendix Table S1 in Supplemental Materials found at https://doi.org/10.1016/j.jval.2021.07.002. Most studies considered only 1 novel test, except Sathianathen et al^28^ who compared PHI, the 4Kscore, ExoDx Prostate(IntelliScore), and SelectMDx.^22^^,^^28^ Of the 11 studies comparing different biomarkers, 9 referred to TRUS-guided biopsy to confirm diagnosis, 1 to multiparametric MRI,^22^ and 1 did not report the biopsy method assumed.^23^ Only 3 studies^23^^,^^24^^,^^27^ that compared biomarkers modeled repeat PSA/biomarker testing, assuming annual^24^ or 4-yearly screening.^23^^,^^27^ These intervals were chosen in accordance with the American Urological Association 2009 recommendations (annual screening for men aged 40 years and older with shared decision making)^29^ and the screening protocol used in ERSPC (4 yearly).^7^

#### Strategies compared—biopsy methods

Men with a suspicion of prostate cancer indicated by a PSA test or other biomarker are generally referred for a TRUS-guided biopsy. The different biopsy methods the 7 studies identified compared included MRI-targeted methods and template mapping biopsy.^30^ The definitions of biopsy methods are given in Appendix Table S2 in Supplemental Materials found at https://doi.org/10.1016/j.jval.2021.07.002. Different strategies were compared, including using MRI to decide whether a TRUS-guided biopsy is necessary and to target biopsy and strategies starting with TRUS-guided biopsy and using MRI to decide whether a repeat biopsy is necessary. A total of 3 of the studies comparing biopsy methods^31^, ^32^, ^33^ modeled repeat screening, assuming that men would be screened every 2 years based on the 2013 American Urological Association guideline^34^ or every 4 years based on the ERSPC protocol.^7^

#### Strategies compared—follow-up strategies in men with negative biopsies

A total of 3 studies^35^, ^36^, ^37^ compared follow-up strategies for men with raised PSA and negative MRI, negative prostate biopsy or negative MRI and negative biopsy. The strategies included various biomarkers (PSA, PSA velocity, PSA density, % free PSA, PSA doubling time, PSA density in transition zone, PCA3, PHI) and MRI techniques.

### Model Inputs

#### Accuracy data

All but 6 studies^24^^,^^27^^,^^33^^,^^36^^,^^38^^,^^39^ explicitly reported the sensitivity and specificity of the tests. The assumed sensitivity of a standard biopsy ranged from 0.9 based on ERSPC data^23^^,^^40^ to 0.46 based on de Rooij et al.^28^^,^^41^^,^^42^ The biomarkers were generally assumed to be either particularly sensitive, that is, good at correctly identifying those with the disease, or particularly specific, that is, good at correctly identifying those without the disease. PHI at a threshold of 20, for example, had the highest reported sensitivity (1, but specificity of 0.08) and also the highest reported specificity (0.974, but sensitivity of 0.129).^20^^,^^43^ The MRI-targeted biopsy methods generally had a better balance of sensitivity and specificity, ranging from a sensitivity of 0.965 (specificity of 0.597) for MRI using a Prostate Imaging-Reporting and Data System threshold of ≥3^32^^,^^44^ to 0.770 (specificity 0.68) using fusion biopsy.^28^^,^^45^^,^^46^ Appendix Table S3 in Supplemental Materials found at https://doi.org/10.1016/j.jval.2021.07.002 details the accuracy estimates used along with their evidence sources.

#### Quality of life

As detailed in Table 2, all but 3 studies assigned disutilities to various aspects associated with testing including screening attendance, the biopsy procedure, diagnosis of cancer, treatment, active surveillance, advanced or metastatic cancer, posttreatment or recovery, adverse events associated with biopsy and treatment, and palliative therapy. A total of 9 studies^6^^,^^19^^,^^23^^,^^25^^,^^26^^,^^32^^,^^33^^,^^36^^,^^46^ sourced all utility estimates used in their model from Heijnsdijk et al^6^ who in turn obtained their utility estimates from the Cost-Effectiveness Analysis Registry and various additional studies.^53^, ^54^, ^55^, ^56^, ^57^, ^58^, ^59^, ^60^, ^61^, ^62^, ^63^, ^64^, ^65^ The other studies sourced their utility estimates from various unrelated publications, also in different countries and settings. Where utility estimates were sourced from studies directly measuring health-related QOL, the most common methods used were standard gamble^56^^,^^67^^,^^68^ and time trade-off^69^^,^^70^ and the most common instruments used were EQ-5D^53^^,^^71^, ^72^, ^73^ and 12-item Short Form Medical Survey.^74^^,^^75^ None of the included studies provided the Ara et al^76^ recommended level of detail on health state utility values that are sourced from the literature, that is, detail on searches, inclusion/exclusion criteria, the quality and relevance of included studies, and a justification for the utility values chosen. Only 5 studies fully reported the uncertainty in the disutility estimates used.^31^, ^32^, ^33^^,^^41^^,^^42^

**Table 2:** Disutility estimates used for prostate cancer states, tests, and treatments in the identified economic models (annual values)

Study	Biopsy	Diagnosis	RP	RT	AS	Advanced cancer	Posttreatment	AEs	Other	Source	Report uncertainty
Barnett et al^32^	0.006	0.017	0.247	-	0.03	0.3	0.05	0.0161 (postbiopsy infection)	0.0002 (PSA screening) 0.00077 (MRI) 0.60 (palliative therapy)	^6^^,^^47^	Yes
Cerantola et al^38^	-	-	-	-	-	-	0.08	-	0.22 (relapse)	^48^	No
de Rooij et al^41^	-	-	0.33	0.27	0.16	-	-	-	-	^56^	Yes
Dijkstra et al^25^	0.006	0.017	0.228	0.247	0.03	-	0.05	-	-	^6^	No
Faria et al^30^	0.007 (TPM biopsy)	-	-	-	-	0.137	-	-	-	^47^, PROMIS IPD ^4^^,^^71^	Only for TPM biopsy
Govers et al^19^^,^^26^	0.006	0.017	0.228	0.247	0.03	-	0.05	-	-	^6^	No
Heijnsdijk et al^23^	0.006	0.017	0.247	0.228	0.03	0.3	0.05	-	0.0002 (screening attendance) 0.60 (palliative therapy)	^6^	No
Mowatt et al^37^	-	-	-	-	-	0.365	-	0.16 (urinary incontinence) 0.17 (bowel problem) 0.12 (erectile dysfunction)	0.11 (localized [undiagnosed]) 0.1 (localized [diagnosed]) 0.19 (locally advanced [undiagnosed]) 0.18 (locally advanced [diagnosed])	^66^^,^^68^^,^^69^	Only for cancer states
NICE Guideline^35^	0.004, 0.007 (template mapping biopsy)	-	-	-	-	0.137	-	-	0.027 (low risk) 0.029 (intermediate risk) 0.027 (high risk)	^6^^,^^47^^,^^49^^,^^71^^,^^72^	No
Nichol et al^24^	0.027	-	-	-	-	-	-	-	0.2 (PCa)	^50^^,^^51^^,^^56^	Only for PCa
Nicholson et al^36^	0.006	-	-	-	-	-	-	-	-	^6^	No
Pahwa et al^42^	0.027	Only lifetime QALYs reported								^50^	Yes
Sathianathen et al^28^	0.004	-	0.14	-	0.03	0.42	0.05	-	-	^6^^,^^50^^,^^67^	Yes
Venderink et al^46^	0.006	0.02	0.25	0.23	0.03	0.55	0.05	-	-	^6^	No
Barnett et al^31^	0.00577	0.0167	0.247	-	0.03	0.3	0.05	0.0161 (postbiopsy infection)	0.0002 (PSA screening) 0.00077 (PET/mpMRI) 0.60 (palliative therapy)	^6^^,^^47^^,^^52^	Yes
Callender et al^33^	-	-	-	-	-	-	-	-	0.07 (PCa)	^2^	Yes
Teoh et al^39^	0.027								0.2 (PCa)	^24^^,^^51^^,^^56^^,^^61^	No
Karlsson et al^37^	0.1	0.2	0.33 (part 1), 0.23 (part 2)	0.27 (part 1), 0.22 (part 2)	0.03	0.6	0.05		0.60 (palliative therapy), 0.01 (PSA test)	^6^	No

*Note.* “-”, not applicable as disutility not applied in model.

AE indicates adverse event; AS, active surveillance; IPD, individual participant data; mpMRI, multiparametric magnetic resonance imaging; MRI, magnetic resonance imaging; NICE, National Institute for Health and Care Excellence; PCa, prostate cancer; PET, positron emission tomography; PROMIS, Patient-Reported Outcomes Measurement Information System; PSA, prostate-specific antigen; QALY, quality-adjusted life-year; RP, radical prostatectomy; RT, radiotherapy; TPM, transperineal mapping.

#### Resource use

Most studies took a healthcare provider perspective for the analysis (only including costs incurred to the provider rather than any wider patient or societal costs). A total of 2 studies stated that a societal perspective was taken but did not detail the societal costs that were included.^23^^,^^24^ Another 2 studies included productivity costs in terms of missed days of work when a patient undergoes a test or treatment.^27^^,^^42^ No study gave a justification for the perspective taken. The main costs included were the cost of testing, biopsy, and subsequent management strategy. Thirteen studies included costs of complications arising from biopsy.^19^^,^^20^^,^^22^^,^^25^^,^^26^^,^^30^, ^31^, ^32^^,^^35^, ^36^, ^37^^,^^42^^,^^46^ Only 7 studies explicitly stated that costs associated with complications arising from treatment were included.^19^^,^^25^^,^^26^^,^^30^^,^^35^^,^^37^^,^^46^

### Modeling Methods

#### Model type

Table 3 details model characteristics including model type, time horizon, and cycle length. A total of 8 combined decision tree/Markov cohort models were identified. In 5 of these, the decision tree reflected the diagnostic process and the Markov model reflected treatment.^26^^,^^30^^,^^39^^,^^41^ In the others, the decision tree captured both diagnosis and treatment and the Markov model was used for posttreatment states.^25^^,^^28^^,^^46^ The treatment allocation assumed in the studies that modeled this is shown in Appendix Table S4 in Supplemental Materials found at https://doi.org/10.1016/j.jval.2021.07.002. A total of 8 cohort Markov models,^21^^,^^24^^,^^31^, ^32^, ^33^^,^^35^^,^^37^^,^^38^ 2 continuous time discrete-event microsimulation models (the MIcrosimulation SCreening Analysis model)^23^ and the Prostata model,^27^ and 4 decision tree models^20^^,^^22^^,^^36^^,^^42^ were also identified. No study provided a justification for choosing one model type over another.

**Table 3:** Model characteristics

Study	Model type	Progression modeled	Health states in Markov model	Definition of low-risk cancer	Definition of intermediate risk cancer	Definition of high-risk cancer	Time horizon	Cycle length	DSA	PSA
Dijkstra et al^25^	Decision tree/Markov	No	High-grade PCa, low-grade PCa, missed PCa	G ≤ 6	-	G ≥ 7	18 years	1 year	Yes	No
Sathianathen et al^28^	Decision tree/Markov	No	NR	-	-	-	Lifetime	6 months	Yes	Yes
Govers et al^26^	Decision tree/Markov	No	High-grade PCa, low-grade PCa, missed PCa	G ≤ 6	-	G ≥ 7	Lifetime	1 year	Yes	No
Faria et al^30^	Decision tree/Markov	Yes	Progression free, metastatic	PSA < 10, G < 6	PSA 10-15 or G7	G > 8	Lifetime	NR	Yes	Yes
Venderink et al^46^	Decision tree/Markov	No	Status after prostatectomy, status after radiotherapy, status after active surveillance	-	-	-	18 years	1 year	Yes	No
de Rooij et al^41^	Decision tree/Markov	No	Alive, dead	G3 + 3 or small size 3 + 4	-	Large tumors with a G3 + 3 or ≥3 + 4	10 years	1 year	Yes	No
NICE Guideline^35^	Decision tree/Markov	Yes	Low risk, intermediate, high risk, metastatic	G ≤ 6, PSA ≤ 10	G = 7 or 10 ≤ PSA < 20	G ≥ 8 and PSA > 20	Lifetime	3 months	Yes	Yes
Nichol et al^24^	Markov cohort	No	Alive, dead	-	-	-	Lifetime	1 year	Yes	Yes
Schiffer et al^21^	Markov cohort	No	NR	-	-	-	Up to treatment	NR	Yes	Yes
Barnett et al^32^	Markov cohort	Yes	G < 7, G = 7, G > 7, extraprostatic or lymph node positive	G < 7	G = 7	G > 7	Until death	1 year	Yes	No
Cerantola et al^38^	Markov cohort	No	MRGTB/TRUSGB; follow-up of PCa-naive patients with DRE, PSA, and TRUSGB as required; low-risk PCa; intermediate/high-risk PCa; active surveillance; curative-intended treatment; biochemical recurrence after curative treatment; metastatic/castration-resistant PCa	-	-	-	5, 10, 15, and 20 years	1 year	Yes	No
Mowatt et al^37^	Markov cohort	Yes	Localized (T1-T2) (low risk); localized (intermediate risk); localized (high risk); locally advanced (T3); metastatic	G ≤ 6, PSA ≤ 10, ≤ T1a	G ≤ 7, PSA ≤ 20, ≤ T2b	G > 7, PSA > 20, > T2b	30 years	3 months	Yes	Yes
Pahwa et al^42^	Decision tree	No	-	G ≤ 6	-	G ≥ 7	Until death	-	Yes	No
Nicholson et al^36^	Decision tree	No	-	-	-	-	3 years	-	Yes	Yes
Bouttell et al^20^	Decision tree	No	-	-	-	-	Up to biopsy	-	Yes	Yes
Heijnsdijk et al^23^	Microsimulation	Yes	T1 G < 7, G = 7, G > 7; T2 G < 7, G = 7, G > 7; T3+ G < 7, G = 7, G > 7, each state can be local or metastatic	-	-	-	Lifetime	-	Yes	No
Barnett et al^31^	Markov cohort	Yes	G < 7, G = 7, G > 7, extraprostatic or lymph node positive	G < 7	G = 7	G > 7	Until death	1 year	Yes	No
Callender et al^33^	Markov cohort	No	Healthy, PCa	-	-	-	Lifetime	1 year	Yes	Yes
Kim et al^22^	Decision tree	No	-	-	-	-	Up to diagnosis	-	Yes	No
Teoh et al^39^	Decision tree/Markov	No	PCa, no PCa	-	-	-	25 years	1 year	Yes	Yes
Karlsson et al^27^	Microsimulation	Yes	T1-T2 G < 7, G = 7, G > 7; T3+ G < 7, G = 7, G > 7; Metastatic G < 7, G = 7, G > 7,	-	-	-	Lifetime	-	Yes	Yes
Govers et al^19^	Decision tree/Markov model	No	Treatment, no treatment, delayed treatment	G ≤ 7		G≥7	18 years	1 year	Yes	No

*Note.* “-”, not included in the model.

DRE indicates digital rectal examination; DSA deterministic sensitivity analysis; G, Gleason grade; MRGTB, magnetic resonance imaging-guided transrectal ultrasound biopsy; NR, not reported; PCa, prostate cancer; PSA, probabilistic sensitivity Analysis; TRUSGB, transrectal ultrasound-guided guided biopsy.

The decision trees generally used data on disease prevalence and accuracy of the tests to categorize men into true positives, false positives, true negatives, and false negatives^20^^,^^25^^,^^46^ with some also incorporating the clinical significance of cancer.^25^^,^^26^^,^^30^^,^^41^^,^^42^ The Markov models captured cancer progression and survival. All but 4 studies developed a de novo model.^23^^,^^27^^,^^32^^,^^33^ Cycle length varied from 3 months to 1 year. The only study that reported a justification for the cycle length chosen was the NICE guideline, which stated that the guideline development committee confirmed that a cycle length of 3 months is sufficient to reflect possible clinical events a person with prostate cancer may experience.^35^

#### Sensitivity analyses

All studies conducted a deterministic sensitivity analysis where input parameters or sets of parameters were varied to see the impact on results. Half (11 of 22) of the studies also performed a probabilistic sensitivity analysis where repeated simulations sampled all parameters from their respective distributions to observe the impact on results.^24^^,^^27^^,^^28^^,^^30^^,^^33^^,^^35^, ^36^, ^37^^,^^39^^,^^41^^,^^42^ No study performed a Value of Information analysis to determine the value of further research in prostate cancer screening.^69^

#### Model structure

The structure of a model relates to how different health states are categorized and how patients move between health states. Related to this, the natural history of a disease refers to how a disease progresses in a person over time in the absence of treatment.^70^ Only 7 of the included models^23^^,^^27^^,^^30^, ^31^, ^32^^,^^35^^,^^37^ took account of how prostate cancer progresses through different health states and how the introduction of a new test might affect this, and all of these captured this progression differently. The health states included in the models are shown in Table 3. Heijnsdijk et al^23^ and Karlsson et al^27^ used the most detailed breakdown with both T-stage and Gleason grade modeled. In comparison, the only health states modeled in Faria et al^30^ were progression free and metastatic cancer. A total of 8 studies modeled survival time from diagnosis only,^19^^,^^25^^,^^26^^,^^33^^,^^38^^,^^39^^,^^41^^,^^46^ with no progression through health states. A total of 4 did not model beyond diagnosis.^14^^,^^16^^,^^22^ In addition, the definition of clinically significant cancer varied across studies (Table 3). Of all the models, 6 did not consider stages or grade of cancer, only the presence or absence of cancer.^24^^,^^26^^,^^28^^,^^33^^,^^39^^,^^46^

#### Reporting of overdiagnosis and mechanism of screening benefit

Overdiagnosis and overtreatment owing to the identification of cancers that would never progress to cause prostate cancer related death or illness in a man’s lifetime are key factors to consider when testing men for prostate cancer. Only 3 studies^23^^,^^27^^,^^33^ provided estimates of the impact of screening on overdiagnosis. Both Heijnsdijk et al^23^ and Karlsson et al^27^ defined overdiagnosed cancers as additional cancers detected through screening that were not detected in the “no screening” arm. Heijnsdijk et al^23^ estimated that 5% fewer overdiagnosed cancers would be detected through the use of PHI compared with PSA, and Karlsson et al^27^ predicted 15% fewer overdiagnosed cancers through the use of Stockholm3 when PSA values were above 2 ng/mL compared with PSA alone. Callender et al^33^ estimated age-specific overdiagnosis by multiplying the number of cases by an equation derived from Pashayan et al,^77^ defined as the probability that a PSA-detected case would have taken longer than the remaining lifetime to progress to clinical cancer. They found that MRI-first risk-stratified screening was associated with a 10.4% to 72.6% lower probability of overdiagnosis in screen-detected cases, depending on the 10-year absolute risk thresholds at which individuals were eligible for screening.

In addition, different approaches to measuring the benefit of screening in nonoverdiagnosed men are possible and the choice of method may affect results. Stage-shift screening models assume that the benefit associated with screening is due to a shift to a less advanced stage at diagnosis resulting in improved survival. Cure models assume that if cancers are detected earlier they can be treated and that curative treatment has the potential to prevent cancer-specific mortality.^78^ Only 1 study, Heijnsdijk et al,^23^ explicitly stated that the assumed mechanism of benefit of screening in their model was as a cure proportion, which assumes that a percentage of men are cured owing to screening and therefore avoid a death from prostate cancer. The other studies did not consider overdiagnosis nor give any detail on the mechanism of benefit of screening assumed.

### Cost-Effectiveness Results

To aid comparison, all reported costs were inflated to the 2020 price year and converted to US dollars, taking purchasing power parities between countries into account. This was done using the web-based tool developed by the Campbell and Cochrane Economics Methods Group and the Evidence for Policy and Practice Information and Coordinating Center.^79^ In reality, the costs are not comparable because different countries have different healthcare systems, care pathways, and negotiated prices. Therefore, original costs are also reported.

#### Biomarkers

Of the 11 studies that compared PSA testing with testing with a new biomarker, 6 studies found that introducing the new biomarker saves costs and increases QALYs^19^^,^^24^, ^25^, ^26^^,^^28^^,^^39^ (Table 4). A total of 3 did not measure QALYs but found that diagnostic costs were reduced,^20^, ^21^, ^22^ and one found that the introduction of a new test increased both costs and QALYs.^27^ Of the studies that considered progression through stages or grades of cancer, Heijnsdijk et al^23^ found that PSA + PHI testing saves costs compared with PSA testing alone and results in the same QALYs^23^ and Karlsson et al^27^ estimated an incremental cost-effectiveness ratio (ICER) of €5663 for screening using Stockholm3 when PSA values were above 2 ng/mL compared with PSA alone. The results from all studies were generally driven by a decrease in negative biopsies.

**Table 4:** Cost-effectiveness results from studies; where >2 interventions were compared, the ICER for the most cost-effective intervention is presented.

Author	Tests compared	Difference in costs∗	Difference in QALYs†	ICER	Probability cost-effective
Bouttell et al^20^	PHI vs PSA	−HK$5500 (−$943)	NA	NA	NR
Heijnsdijk et al^23^	PHI vs PSA	-€33 (-$47)	0	NA	NR
Nichol et al^24^	PHI vs PSA	−$201 to −$1199 (−$243 to −$1447)	0.01-0.08	Dominates	77%-70% or 78%-71% % at a range of $0-$200 000 WTP using PSA thresholds ≥2 ng/mL and ≥4 ng/mL, respectively
Govers et al^26^	SelectMDx vs PSA	−$1694 (−$1854)	0.045	Dominates	NR
Dijkstra et al^25^	SelectMDx vs PSA	−€128 (−$170)	0.025	Dominates	NR
Schiffer et al^21^	UPA-PC vs PSA	−€297 (−$440)	NA	NA	NR
Kim et al^22^	MRI + biopsy only if PHI ≥ 30 vs MRI + biopsy for all	−£191 (−$280)	NA	NR	NR
Teoh et al^39^	PHI vs PSA	$4562 (−$4657)	0.35	Dominates	NR
Karlsson et al^27^	Stockholm3 if PSA > 2 ng/mL vs PSA	€14 ($18)	1	€5663 ($7082)	97% at WTP €50 000
Govers et al^19^	SelectMDx vs PSA	France: −€1217 (−$1620) Germany: −€439 (−$605) Italy: −€757 (−$1089) Spain: −€247 (−$405)	France 0.036 Germany 0.026 Italy 0.043 Spain 0.028	Dominates	NR
Venderink et al^46^	MRI TRUS fusion biopsy vs TRUS-guided biopsy	€175 ($236)	0.1263	€1386 ($1869)	NR
Cerantola et al^38^	MRI cognitive-targeted biopsy vs TRUS-guided biopsy	−CAD$2187 (−$1960)	0.168	Dominates	NR
de Rooij et al^41^	MRI-targeted biopsy vs TRUS-guided biopsy	€31 ($42)	0.10	€323 ($442)	80% at WTP higher than €2000
Faria et al^30^	mpMRI guided biopsy vs TRUS-guided biopsy	NR	NR	£7076 ($10 519)	NR
Pahwa et al^42^	MRI cognitive-targeted biopsy vs TRUS-guided biopsy	−$1771 (−$1882)	0.198	Dominates	94.05% at WTP $50 000 and 93.9% at WTP $100 000
Mowatt et al^37^	T2-MRI vs TRUS-guided biopsy	£7 ($12)	0.00054	£12 315 ($21 013)	34% at WTP £30 000
Barnett et al^32^	Combined (standard + targeted fusion) biopsy vs TRUS-guided biopsy	NR	NR	$23 483 ($24 340)	NR
Nicholson et al^36^	clinical assessment + mpMRI vs clinical assessment	£113 449 ($180, 497)	3.35	£33 911 ($53 952)	100% at WTP £37 000
Barnett et al^31^	hybrid 18F-choline PET/mpMRI with Likert scoring vs TRUS-guided biopsy	NR	NR	$35 108 ($35 841)	NR
Callender et al^33^	MRI-first risk-stratified screening at 10-year absolute risk threshold of 7.5% vs no screening	£28 ($35)	0.0042	NR	NR

ICER indicates incremental cost-effectiveness ratio; mpMRI, multiparametric magnetic resonance imaging; MRI, magnetic resonance imaging; NA, not available; NR, not reported; PET, positron emission tomography; PHI, Prostate Health Index; PSA, prostate-specific antigen; QALY, quality-adjusted life-year; TRUS, transrectal ultrasound; UPA-PC, urinary proteome analysis for prostate cancer diagnosis; USD, US dollars; WTP, willingness to pay.

∗ Costs are in reported currency with USD 2020 costs in brackets to aid comparison.

† NA indicates not applicable because the study was not a cost-utility analysis. NR indicates not reported because the study did not report differences between interventions.

#### Biopsy methods

A total of 7 of the 8 studies that compared MRI-guided biopsy strategies to each other and to TRUS-guided biopsy found at least 1 MRI-guided strategy to be cost-effective (increased costs but also increased QALYs). The exception was Cerantola et al^38^ who found that MRI-guided biopsy dominated TRUS-guided biopsy (reduced costs and increased QALYs). ICERs for MRI-guided biopsy methods compared with standard methods ranged from €323 per QALY in a study conducted from a The Netherlands perspective^41^ to $35 108 per QALY in a US study,^31^^,^^32^ both indicating cost-effectiveness according to the generally accepted cost-effectiveness thresholds in the respective countries.^80^^,^^81^ The increased QALYs and reduced costs were generally owing to an avoidance of the adverse effects and resource use associated with overdiagnosis.

#### Follow-up strategies

A total of 2 of the studies comparing follow-up strategies in men with a previous negative biopsy did not identify a clear indication of cost-effectiveness for any strategy. The NICE guideline^35^ concluded that PSA velocity, density, and % free PSA may be the best indicators to trigger further diagnostics in higher risk populations, however the “no screening” strategy seemed optimal for the lowest-risk subpopulation who had MRI Likert scores of 1 or 2 (very unlikely/unlikely that the patient has prostate cancer that needs to be treated) and 2 previous negative biopsies. Nicholson et al^36^ found no strategy to be cost-effective. Mowatt et al^37^ found the base-case ICER for T2-MRI to be below the UK willingness to pay threshold (£30 000 per QALY) for all cohorts modeled.

#### Assessing uncertainty in cost-effectiveness results

A total of 5 studies found that the results were sensitive to the potential of the tests to identify cancer, particularly clinically significant cancer.^25^^,^^26^^,^^30^^,^^33^^,^^41^^,^^42^ A total of 3 studies found results to be sensitive to the assumed prevalence of cancer and significant cancer.^41^^,^^42^^,^^46^ The cost of the tests was also stated as an important factor in 5 of the studies.^20^^,^^27^^,^^28^^,^^30^^,^^46^ Furthermore, studies found results to be sensitive to probabilities of cancer progression in undiagnosed cases,^31^^,^^32^^,^^35^ increasing or decreasing survival rates in men treated for prostate cancer,^35^^,^^46^ and QOL values used for diagnosed cancer states.^27^^,^^31^^,^^32^^,^^37^ For example, the NICE guideline^35^ stated that increasing the survival rate resulted in the strategy where all men receive an immediate TRUS and no subsequent follow-up to be optimal in the majority of subpopulations. In contrast, Venderink et al^46^ found that if the yearly survival rate among patients with treated clinically significant prostate cancer were to decrease from 98.6% to 93.2%, TRUS-guided biopsy would be the most cost-effective strategy. Mowatt et al^37^ assessed the impact of applying a utility decrement of 0.035 (half of the disutility associated with having moderate anxiety rather than no anxiety on the EQ-5D) to patients with undiagnosed cancer, to reflect potential disutility from increased anxiety associated with having a high PSA but no diagnosis. This resulted in systematic TRUS being the most cost-effective intervention.

### Quality of Included Studies

The overall mean percentage of the applicable Consolidated Health Economic Evaluation Reporting Standards criteria met by each study was calculated at 71%, with a range of 37% to 100% and a median of 68%. Only 1 study satisfied all applicable criteria (scoring 100%) (Appendix Table S5 in Supplemental Materials found at https://doi.org/10.1016/j.jval.2021.07.002). Risk of bias was assessed using the Bias in Economic Evaluation checklist. No studies had bias in terms of comparators or ordinal ICERs. Partial biases related to structural assumptions, type of model used, and data identification occurred in most studies (Appendix Table S6 in Supplemental Materials found at https://doi.org/10.1016/j.jval.2021.07.002).

## Discussion

This review aimed to identify economic models evaluating new diagnostic tests for prostate cancer; determine the evidence base and cost-effectiveness results, provide an overview of the characteristics of these models and their data sources to aid in the development of future cost-effectiveness models in this area, and assess the limitations of available models, providing guidance on future improvements.

A total of 22 studies were identified, all published between 2011 and 2021. A total of 11 compared the cost-effectiveness of new urinary or blood biomarkers with each other or with the standard of care (a PSA test). Another 8 compared different approaches with prostate biopsy and 3 compared follow-up strategies in men who have a negative initial biopsy result. Most models used either a combined decision tree/Markov or purely Markov model structure with only 7 modeling progression through stages or grades of cancer. Substantial variability was seen in the model pathways of prostate cancer natural history, the data sources used to inform progression, treatment allocation assumed for high- and low-risk cancers, disutility values assigned to health states, and the assumed accuracy of the tests. All but 1 study^36^ found the introduction of these novel tests to be cost-effective; nevertheless, in some cases, the benefits may be overestimated because of a failure to take account of overdiagnosis and the natural history of the disease in untested men.

### Limitations of Included Models

The studies identified had several key limitations. Although they compared novel tests to diagnose prostate cancer, many failed to take into account the complexity of the disease, including stage or grade of cancer and how cancer progresses in diagnosed and undiagnosed cases. This calls into question the reliability of the results given that the cost-effectiveness of a new test may be overestimated if the cancers it identifies would never progress to cause symptoms or mortality if not identified. The purpose of screening and testing is to identify cancers at an early stage when they are more amenable to treatment. If cost-effectiveness models do not differentiate between cancer stages, it is difficult to measure the effects of early diagnosis.^13^

Only half of the studies performed a probabilistic sensitivity analysis to fully account for the uncertainty in the model parameters. Of the 19 studies which included QALYs, 9 cited Heijnsdijk et al^6^ as the source for their utility estimates who in turn obtained their estimates from studies in various countries and settings and using different evaluation techniques. Although this approach indicates that there is likely no alternative common source for these utility parameters, this is against best practice because the values cannot be considered to be equivalent when measured in different populations.^82^ Notably, 13 of the 19 studies did not report uncertainty in their QALY estimates, suggesting that this uncertainty was not accounted for.^23^^,^^25^^,^^26^^,^^35^^,^^36^^,^^38^^,^^46^ Given that QALY estimates can often have a substantial impact on the intervention considered most cost-effective, it is important that any underlying uncertainty in these estimates is fully accounted for.

A total of 3 studies used a time horizon of 3 years or less, modeling only up to biopsy, which is unlikely to be long enough to capture the impact of timely and accurate diagnosis of prostate cancer, because of its long-term nature.^20^^,^^21^^,^^36^ Although most of the models represented the entire diagnostic pathway from test to treatment, the majority of these compared either new biomarkers or new MRI-guided biopsy methods with few comparing combinations of tests. Although both biomarker and imaging advancements are important, it seems worthwhile for them to be considered in combination given that this is how they may be used in practice.^83^^,^^84^

### Recommendations for Future Cost-Effectiveness Models

Any future model should consider the entire diagnostic pathway, which may include both new biomarkers and biopsy methods, to comprehensively assess the “true” cost-effectiveness of these tests within a diagnostic strategy for prostate cancer. When modeling the lifetime cost-effectiveness of a test to diagnose prostate cancer, it is important to consider the natural history of the disease and how a test may affect this, to ensure that the benefit of the test is accurately represented and overdiagnosis is considered. The studies identified in this review that modeled the natural history of prostate cancer all did so in different ways, suggesting a lack of clarity in the field. Any future model should consider this carefully with the help of clinical experts.

One potential approach to overcome future model disparity is comparative modeling, an approach taken by the Cancer Intervention and Surveillance Modeling Network, which uses statistical/simulation modeling to examine the impact of screening on cancer incidence and mortality. With comparative modeling, the same or a similar set of inputs is used across a range of models with all models then reporting the same intermediate and final outputs.^85^ An approach such as this could be beneficial in the area of reflex testing in prostate cancer to enable a truer comparison between strategies.

Although a formal literature review to identify health state utility values has not been performed, the values used in previous models indicate a potential paucity of information on how prostate cancer treatment and adverse effects affect QOL. This should be considered carefully and uncertainty fully accounted for where it exists, given that this could greatly affect the results of a cost-utility model.

### Strengths and Limitations of Review

The strength of this systematic review is that it has provided an overview of cost-effectiveness models published in the last 10 years, which have compared novel diagnostic methods in prostate cancer. It has offered insight into the data parameters that will be needed to populate a future cost-effectiveness model incorporating new tests and diagnostic strategies in prostate cancer and potential sources of information for these parameters. It has also highlighted the limitations of previous models. The results from the review have emphasized the importance of accurately estimating factors such as the sensitivity of tests, the prevalence of disease, and the progression of the disease.

A limitation is that this review cannot provide recommendations on the most cost-effective test or diagnostic strategy because the studies are too heterogenous for the cost-effectiveness results to be compared. A further limitation is that, although the systematic review did not identify any relevant studies published between 2009 and 2011, the 2009 cutoff could potentially miss economic models of novel diagnostic methods published before 2009. Furthermore, 100% double screening and data extraction were not feasible because of a lack of resources. In line with guidelines, checks for accuracy were performed by comparing categorization of studies and data extraction with those of a second reviewer who independently screened 10% of randomly selected titles and abstracts and performed data extraction on 20% of the included studies.^16^^,^^17^^,^^86^

### Comparison With Previous Reviews

This is the first study to identify cost-effectiveness models focused on screening and diagnostic strategies beyond standard PSA-based testing. One recent systematic review assessed model-based economic evaluations of PSA-based screening strategies only.^82^ This review also found a significant variation in model pathways to reflect cancer progression in the 10 included studies and limited and heterogenous evidence on QOL. A total of 3 older reviews were also identified but all assessed PSA-based screening only.^87^, ^88^, ^89^

## Conclusion

The introduction of new biomarkers and MRI-guided biopsy methods in the studies identified in this review has been shown to lead to an improvement in health outcomes and a decrease or acceptable increase in costs.^20^^,^^25^^,^^26^ Current concerns around implementing PSA-based prostate cancer screening strategies are due to overdiagnosis and overtreatment,^90^ and these newer methods may lead to a reduction in these factors. This review has highlighted the substantial complexity involved in modeling the cost-effectiveness of diagnostic tests in prostate cancer to determine whether these strategies should be used at all and, if so, how and in what combination. To ensure the cost-effectiveness of any diagnostic strategy is assessed robustly, there is a need to ensure that disease progression in diagnosed and undiagnosed cases is accurately represented, uncertainty is fully accounted for, QOL estimates are measured as accurately as possible, and the possibility of repeat screening and testing in men with a negative diagnosis is considered.

## Article and Author Information

**Author Contributions:***Concept and design*: Keeney, Thom, Turner, Martin, Sanghera

*Acquisition of data*: Keeney, Morley

*Analysis and interpretation of data*: Keeney, Thom, Turner, Martin, Sanghera, Morley

*Drafting of the manuscript*: Keeney, Thom, Turner, Martin, Sanghera, Morley

*Critical revision of the paper for important intellectual content*: Keeney, Thom, Turner, Martin, Sanghera

*Statistical Analysis*: Keeney

*Obtainingfunding:* Martin

*Supervision*: Thom, Martin, Sanghera

**Conflict of Interest Disclosures:** Ms Keeney and Dr Thom reported performing commercial consulting for Novartis Pharma AG, Roche, Pfizer Inc, and Bristol-Myers Squibb outside the submitted work. Dr Thom reported performing commercial consulting for Janssen outside the submitted work and receiving a covered university salary from the National Institute for Health Research Biomedical Research Centre at University Hospitals Bristol NHS Foundation outside the submitted work. Dr Martin reported receiving grants from Cancer Research UK to evaluate the long-term effectiveness and cost-effectiveness of population-based screening and treatment for prostate cancer: the CAP and ProtecT randomized controlled trials, during the conduct of the study, and being supported by the National Institute for Health Research Biomedical Research Centre at University Hospitals Bristol and Weston NHS Foundation Trust and the University of Bristol outside the submitted work. Dr Turner reported receiving a grant from Cancer Research UK during the course of this study. No other disclosures were reported.

**Funding/Support:**10.13039/501100000289Cancer Research UK and the United Kingdom Department of Health (C11043/A4286, C18281/A8145, C18281/A11326, C18281/A15064, and C18281/A24432). The views and opinions expressed by authors in this publication are those of the authors and do not necessarily reflect those of the United Kingdom National Institute for Health Research or the Department of Health and Social Care.

**Role of the Funder/Sponsor:** The funder had no role in the design and conduct of the study; collection, management, analysis, and interpretation of the data; preparation, review, or approval of the manuscript; and decision to submit the manuscript for publication.

## Supplemental Material

Appendices 1-3 and Appendix References
