# Supplementary material for: Systematic Review of Cost-Effectiveness Models in Prostate Cancer: Exploring New Developments in Testing and Diagnosis
Source: Value Health. 2022 Jan;25(1):133–46. doi: 10.1016/j.jval.2021.07.002 (PMC8752463; doi:10.1016/j.jval.2021.07.002)
Supplement: Appendices 1-3 and Appendix References [file mmc1.pdf]

Appendix

Appendix 1: Search terms

Ovid - Medline

1. exp prostatic neoplasms/

2. (cancer adj3 (prostate or prostatic)).tw.

3. (carcinoma adj3 (prostate or prostatic)).tw.

4. (neoplas$ adj3 (prostate or prostatic)).tw.

5. (malignan$ adj3 (prostate or prostatic)).tw

6. (prostat$ adj3 (neoplasm$ or cancer or carcinoma or tumo?r$ or malignan$)).tw

7. 1 or 2 or 3 or 4 or 5 or 6

8. Prostate-Specific Antigen/

9 (prostate specific antigen or prostate-specific antigen or psa) tw

10. Mass screening/

11. (Screen$ or test$) tw

12. 8 or 9 or 10 or 11

13. exp “costs and cost analysis”/

14. (model adj3 (economic or cost)).tw.

15. (cost adj3 (effect$ or util$)).tw.

16. (economic adj3 (anal$ or eval$)).tw.

17. (natural history model) tw

18. (screen$ model$) tw

19. (disease progression model$) tw

20. 13 or14 or 15 or 16 or 17 or 18 or 19

21. 7 and 12 and 20

22. limit 12 to yr=”2009-Current”

Ovid - EMBASE

1. prostatic neoplasms [not a MESH term]

2. exp prostate tumor/ [broader than cancer]

3. (cancer adj3 (prostate or prostatic)).tw.

4. (carcinoma adj3 (prostate or prostatic)).tw.

5. (neoplas$ adj3 (prostate or prostatic)).tw.

6. (malignan$ adj3 (prostate or prostatic)).tw

7. (prostat$ adj3 (neoplasm$ or cancer or carcinoma or tumo?r$ or malignan$)).tw

8. 1 or 2 or 3 or 4 or 5 or 6 or 7

9. Prostate-Specific Antigen/

10 (prostate specific antigen or prostate-specific antigen or psa) tw

11. Mass screening/

12. (Screen* or test*) tw

13. 9 or 10 or 11 or 12

14. exp Economic evaluation/

15. (model adj3 (economic or cost)

16. (cost adj3 (effect$ or util$))

17. (economic adj3 (anal$ or eval$)

18. (natural history model) tw

19. (screen$ model$) tw

20. (disease progression model$) tw

21. 14 or 15 or 16 or 17 or 18 or 19 or 20

22. 8 and 13 and 21

23. limit 12 to yr=”2009-Current”

Cochrane – NHS EED

#1 MeSH descriptor: [Prostatic Neoplasms]

#2 (prostat* NEAR/3 (neoplasm* or cancer or carcinoma or tumor* or tumour* or malignan*)):

#3 screen* or test*

#4 MeSH descriptor: [Mass screening]

#5 (prostate specific antigen or prostate-specific antigen or psa) tw

#6 Prostate-Specific Antigen/

#7 #1 or #2

#8 #3 or #4 or #5 or #6

#9 #7 and #8

#10 limit publication year from 2009 to 2016, in Economic Evaluations

Cochrane - HTA

#1 MeSH descriptor: [Prostatic Neoplasms]

#2 (prostat* NEAR/3 (neoplasm* or cancer or carcinoma or tumor* or tumour* or malignan*)):

#3 screen* or test*

#4 MeSH descriptor: [Mass screening]

#5 (prostate specific antigen or prostate-specific antigen or psa) tw

#6 Prostate-Specific Antigen/

#7 #1 or #2

#8 #3 or #4 or #5 or #6

#9 #7 and #8

#10 limit publication year from 2009 to 2021, in Technology Assessments

Appendix 2: Data extraction form

Data extraction form for systematic review of model-based economic evaluation methods in prostate cancer screening.

**Title**	
**Author**	
**Year**	
**Country**	
**Study objective**	
**Strategies/tests compared**	
**Threshold for a positive result**	
**Frequency of testing/screening**	
**Starting/stopping age**	
**Types of biopsy**	
**Types of treatment**	
**Population – age/ethnicity/prevalence of prostate cancer**	
**Outcome measure**	
**Economic evaluation type**	
**Cost-effectiveness result**	
**Model type**	
**Justification for model type**	
**Model structure (Stage or grade progression)**	
**Evidence source/s for natural history pathway**	
**Cycle Length**	
**Justification for cycle length**	
**Time horizon**	
**Justification for time horizon**	
**Sensitivity analysis (methods for incorporating uncertainty)**	
**Evidence base for health state utility values**	
**Evidence base for costs**	
**Data on accuracy of tests**	
**Software used**	
**Is overdiagnosis/overtreatment reported? If so, how?**	
**VOI (EVPPI or EVSI)**	

Appendix 3: Tables

Table S1 Definitions of biomarkers used in included studies

Biomarker	Definition
Prostate Health Index (PHI)	blood biomarker calculated by a test analyser from the combination of total PSA (tPSA), free PSA (fPSA), and [-2]proPSA assays. It is used to calculate the probability of PCa and as an aid in distinguishing PCa from benign prostatic conditions for men with a borderline PSA test (e.g. PSA 2-10 ng/mL or 4-10 ng/mL) and non-suspicious digital rectal examination.(25)
SelectMDx (SelectMDx; MDxHealth, Inc., Irvine, CA, USA)	urinary molecular biomarker-based risk score developed to identify patients that are at risk of harbouring high-grade PCa (Gleason score ≥7).(26) This risk score is based on the urinary homeobox C6 (HOXC6) and distal-less homeobox 1 (DLX1) mRNA signature in combination with serum PSA level, PSA density, and other clinical risk factors such as age, prior cancer-negative biopsies, DRE, and family history
Urinary Proteome Analysis for PCa diagnosis (UPA-PC)	based on capillary electrophoresis mass spectrometry (CE/MS) and allows proteome analyses of prostatic secretions in first stream urine (first 10–15 mL urine

	fraction) to distinguish patients with positive PSA and/or DRE with PCa from those without PCa. The test simultaneously determines 12 biomarkers combined as a PC-specific multi-biomarker profile.(27)
4Kscore® Test (OPKO Diagnostics, LLC)	incorporates measured blood levels of four kallikrein proteins: total PSA, free PSA, intact PSA, and human kallikrein 2 plus clinical information (age, DRE findings, and a history of prior negative biopsy result) into an algorithm to calculate an individual man's percentage risk (< 1% to > 95%) of having Gleason score ≥ 7 if a prostate biopsy were to be performed.(28)
ExoDx Prostate (IntelliScore) (EPI)	urine exosome gene expression test, which utilizes exosomal RNA expression levels of three genes to predict the likelihood of having high-grade PCa of Grade Group 2 or greater.(29)
PCA3	a segment of noncoding messenger ribonucleic acid (mRNA) from chromosome 9q21–22 that is overexpressed by more than 95% of all PCas tested (30).
PSA density	serum PSA level (ng/mL) divided by volume of the prostate gland (mL)(1)
Stockholm3	blood-based prostate cancer test that analyzes Total PSA, free PSA, HK2, MSMB and MIC1, more than 100 genetic markers as well as age, earlier prostate biopsy, family history of prostate biopsy and use of 5-alfareducase inhibitors. In addition digital rectal examination (DRE) and prostate volume is used on men referred to urologist(2)

Table S2. Definitions of biopsy methods used in included studies

**Biopsy method**	**Definition**
Standard	Transrectal ultrasound guided
Template mapping	Accurately characterises disease status by sampling the entire prostate every 5 mm (4)
Fusion	the patient undergoes a standard transrectal ultrasound guided biopsy but MRI targets from a preceding MRI scan are digitally “fused” to the ultrasound images so that additional cores can also be taken from those locations under ultrasound visualization (33)
Combined	both standard and targeted fusion biopsies are performed during a single biopsy session (25)
Cognitively guided	the patient undergoes a standard transrectal ultrasound guided biopsy, but the operator performs a biopsy on the basis of his or her knowledge of the location of the lesion at MR imaging (27)
In-gantry/In-bore	involves obtaining tissue samples with direct MR imaging guidance while the patient is in the MR imaging gantry and allows direct visualization of the MR imaging target and the needle at the same time (33)
Magnetic resonance spectroscopy imaging (MRSI)	Further to imaging of water and lipids, which is normally performed with MRI, MRS is a technique that provides detail on protons of molecules other than water and lipids. It can give quantitative information on the presence and quantity of metabolites in the prostate which can be used to estimate the presence and aggressiveness of cancer in prostate tissue (31)
Dynamic contrast-enhanced magnetic resonance imaging (DCE-MRI)	dynamically measures a bolus pass of an intravenously administrated MR contrast agent

	through the prostate. Has been shown to be of use in detection and staging of PC within a multiparametric protocol (31)
Diffusion-weighted magnetic resonance imaging (DW-MRI)	evaluates the microscopic mobility of water molecules in<br>tissue. In addition to its value in the detection of cancer DW-MRI has also been shown to be a promising marker of tumour aggressiveness (31)
^{18}F-Choline PET/mpMRI	mpMRI with the addition of 18F-Choline PET. 18F-Choline is a radioactive substance being studied in positron emission tomography (PET) imaging to find certain types of cancer. 18F-choline gets taken up by cells in the body and more of it is taken up by cancer cells than by normal cells. A PET scanner is used to find which cells in the body have taken up 18F-choline. Also called 18F-fluoromethylcholine, 18F-FMCH, and fluorine F 18-fluoromethylcholine.(3)

Table S3. Reported accuracy of tests compared in studies

**Study**	**Test**	**sensitivity**	**type of cancer**	**specificity**	**type of cancer**	**Source**
Dijkstra et al 2017(19)	SelectMDx	0.957	high grade	0.336	low grade	Two prospective multicentre studies in the Netherlands in men who were scheduled for prostate biopsies, based on elevated PSA levels (≥3 ng/ml), abnormal DRE, or a family history of PCa (n=619)(33)
	SelectMDx			0.608	no cancer	
Bouttell et al 2019(17)	PHI cut off 25	0.887	any grade	0.365	any grade	Prospective cohort study of Chinese men with PSA 4-10 ng/mL and non-suspicious DRE (n=569)(34)

	PHI cut off 35	0.613	any grade	0.775	any grade	
	PHI cut off 55	0.129	any grade	0.974	any grade	
Sathianathen et al 2018(22)	TRUS guided biopsy	0.46	low grade			Taken from de Rooij CEA(30)
	TRUS guided biopsy	0.67	high grade			biopsy simulation study (38)
	PHI	0.883	low grade	0.294	no cancer	US multi-center, double-blind, case-control clinical trial in men with no history of PCa, non-suspicious DRE and pre-study PSA of 1.5–11.0 ng/mL (n=1372)(39)
	PHI	0.914	high grade			
	MRI	0.680	low grade	0.51	no cancer	
	MRI	0.880	high grade			
	4k score	0.816	low grade	0.3801	no cancer	US Multi-institutional Prospective Trial in men referred for biopsy (n=1012)(40)
	4k score	0.948	high grade			
	Select MDx	0.830	low grade	0.4	no cancer	Two prospective multicentre studies in the Netherlands in men who were scheduled for prostate biopsies, based on elevated PSA levels (≥3 ng/ml), abnormal DRE, or a family history

						of PCa (n=619)(33)
	Select MDx	0.910	high grade			
	EPI	0.800	low grade	0.3918	no cancer	Prospective multicenter study in the US in men with with PSA levels of 2 to 20 ng/mL (n=255)(41)
	EPI	0.919	high grade			
	MRIGB	0.440	low grade			
	MRIGB	0.910	high grade			
Govers et al 2018(20) Govers et al 2019(4)	SelectMDx	0.957	high grade	0.61	No cancer	Two prospective multicentre studies in the Netherlands in men who were scheduled for prostate biopsies, based on elevated PSA levels (≥3 ng/ml), abnormal DRE, or a family history of PCa (n=619)(33)
		0.660	low grade	0.34	low grade	
Heijnsdijk et al 2016(16)	PSA test	0.790	T1 Gleason 2-6			ERSPC trial (n=42,376)(35)
		0.990	T3 Gleason 8-10			
	Biopsy	0.900				
Schiffer et al 2012(21)	UPA-PC	0.86		0.59		Prospective study carried out in Germany of men with suspicious PSA and/or DRE (n=211)(21)
	Biopsy	0.70		1		

Barnett et al 2018(25)	Standard Biopsy	0.8				Retrospective analysis of 7643 needle biopsies carried out in the US(42)
	Targeted fusion biopsy	0.770	high grade	0.68	high grade	Prospective cohort study of men with elevated PSA or abnormal DRE undergoing both targeted and standard biopsy concurrently at the National Cancer Institute in the US (n=1003)(37)
	Combined biopsy	0.850	high grade	0.49	high grade	
	PI-RADS > 3	0.965	clinically significant	0.597	clinically significant	Prospective study of men who presented for transperineal biopsy after mpMRI in one UK institution (n=201)(36)
	PI-RADS > 4	0.789	clinically significant	0.789	clinically significant	
Pahwa et al 2017(26)	MR Imaging	0.760		0.88		Taken from de Rooij CEA(30)
	Standard Biopsy	0.460				
	MR Cognitive biopsy	0.780	clinically significant			Two retrospective studies, one of 178 men and another of 54 men both undergoing MRI due to elevated PSA levels in Japan (43, 44)

	MR Cognitive biopsy	0.200	clinically insignificant			Systematic review and meta-analysis of 16 studies including 1926 men(45)
	MR fusion biopsy	0.800	clinically significant			Retrospective analysis of men who underwent prebiopsy mpMRI followed by MRI fusion targeted biopsy in one US institution (n=452)(46)
	MR fusion biopsy	0.510	clinically insignificant			Systematic review and meta analysis of 16 studies including 1926 men(45)
	MR guided in-gantry biopsy	0.920	clinically significant			Single-institution, prospective study of biopsy-naive men referred to a urologist with elevated PSA in Australia (n=223)(47)
	MR guided in-gantry biopsy	0.210	clinically insignificant			Systematic review and meta analysis of 16 studies including 1926 men(45)
	All biopsy procedures			1		Assumption
Venderink et al 2017 (28)	mpMRI	0.930	clinically significant	0.21	clinically significant	Prospective cohort study of UK men with clinical suspicion of PCa who underwent mpMRI

							followed by TPM (n=129)(48)
	mpMRI			0.28	any cancer		
	MRI-TRUS fusion	0.770	clinically significant				Prospective cohort study of men with elevated PSA or abnormal DRE undergoing both targeted and standard biopsy concurrently at the National Cancer Institute in the US (n=1003)(37)
	MRI-TRUS fusion	0.500	clinically insignificant				
	MRI-TRUS fusion			1	any cancer		Assumption
	TRUS guided biopsy	0.530	clinically significant				Prospective cohort study of men with elevated PSA or abnormal DRE undergoing both targeted and standard biopsy concurrently at the National Cancer Institute in the US (n=1003)(37)
	TRUS guided biopsy	0.550	clinically insignificant				
	TRUS guided biopsy			1	any cancer		Assumption
de Rooij et al 2014(30)	TRUS guided biopsy	0.456		0.88			Sensitivity: Single institution

						retrospective study (n=438), Single institution prospective study (n=100), Single institution prospective study (n=54), Single institution prospective study (n=71) (49-51) (52)<br><br>Specificity: Single institution prospective study (n=64)(53)
	mpMRI	0.740		0.88		Meta-analysis of seven studies including 526 patients(54)
	MRGB	0.900		1		Assumption
Mowatt et al 2013(31)	TRUS guided biopsy	0.832		1		Prospective study of Italian patients suspected of harbouring PCa after a first negative biopsy (n=340)(55)
	T2-MRI	0.86		0.55		Systematic review and meta-analysis of 15 studies in 620 patients(31)
	MRS	0.92		0.76		Systematic review and meta-analysis of 10 studies in 438 patients(31)
	DCE-MRI	0.79		0.52		Systematic review and

						meta-analysis of 3 studies in 209 patients(31)
	T2-MRI or MRS	0.96		0.31		Systematic review and meta-analysis of 8 studies in 316 patients(31)
	T2-MRI or DCE-MRI	0.88		0.14		Systematic review and meta-analysis of 3 studies in 209 patients(31)
Barnett et al (5)	mpMRI alone (Likert 4–5)	0.8519 (0.6627–0.9581)		0.5517 (0.3569–0.7355)		US prospective single-arm clinical trial (N=63)(6)
	mpMRI alone (PI-RADSv2 3–5)	0.9259 (0.7571–0.9909)		0.5862 (0.3894–0.7648)		
	18F-choline PET/mpMRI (Likert)	0.9259 (0.7571–0.9909)		0.9310 (0.7723–0.9915)		
	18F-choline PET/mpMRI (PI-RADSv2)	0.8889 (0.7084–0.9765)		0.9310 (0.7723–0.9915)		
	Standard biopsy	0.80				Prospective study of autopsy prostates from 164 men who had no history of prostate cancer(7)
	Combined biopsy (targeted biopsy and standard 12-	0.85	Gleason score of ≥ 3 + 4	0.49	Gleason score of ≥ 3 + 4	Prospective cohort study of 1003 men undergoing both targeted and standard biopsy concurrently from 2007 through 2014 at the National Cancer

						Institute in the US (8)
Kim et al (9)	PHI > 20	0.99	≥G2	0.10	≥G2	UK prospective five-centre study (N=545)
	PHI > 20	1.00	≥CPG3	0.08	≥CPG3	
	PHI > 25	0.96	≥G2	0.25	≥G2	
	PHI > 25	0.99	≥CPG3	0.22	≥CPG3	
	PHI > 30	0.92	≥G2	0.40	≥G2	
	PHI > 30	0.95	≥CPG3	0.35	≥CPG3	
	PHI > 35	0.87	≥G2	0.55	≥G2	
	PHI > 35	0.93	≥CPG3	0.49	≥CPG3	
	PSA density > 0.10	0.93	≥G2	0.31	≥G2	
	PSA density > 0.10	0.97	≥CPG3	0.28	≥CPG3	
	PSA density > 0.15	0.81	≥G2	0.51	≥G2	
	PSA density > 0.15	0.90	≥CPG3	0.53	≥CPG3	
	PSA density > 0.20	0.69	≥G2	0.77	≥G2	
	PSA density > 0.20	0.80	≥CPG3	0.72	≥CPG3	

CPG3: Cambridge Prognostic Group 3

Table S4. Treatment allocation assumed (%)

**Study**	**Dijkstra et al(23)**	**Govers et al(25)**	**Barnett et al(22)**	**Pahwa et al(28)**	**Venderink et al(29)**	**Cerantola et al(31)**	**de Rooij et al(32)**	**Barnett et al(5)**	**Govers et al (4) Spain**	**Govers et al (4) Italy**	**Govers et al (4) Germany**	**Govers et al (4) France**	**NICE guideline intermediate risk (high risk in brackets)(16)**
**High Grade/Clinically significant**													
RP	70	54	100	32	70	30	40	100	34	56	67	58	16 (12)
RT	25	40	-	18	25	30	25	-	36	19	18	15	35 (35)

BY	-	-	-	8	-	-	-	-	5				3 (1)
BY+EBRT	-	-	-	-	-	10	-	-	-				-
ADT	-	-	-	33	-	-	-	-	21	19	10	24	-
RT+ADT	-	-	-	-	-	30	-	-	-				-
HT	-	-	-	-	-	-	-	-	-				22 (48)
WW	5	6	-	2	-	-	18	-	4	6	5	4	-
AS	-	-	-	2	5	-	18	-	-				25 (5)
**Low Grade/Clinically insignificant**													
RP	10	50	50	57	40	35	10	50	49	65	50	34	18
RT	10	30	-	7	10	35	-	-	19	11	16	9	20
BY	-	-	-	16	-	15	10	-	17				7
ADT	-	-	-	8	-	-	-	-	5	8	5	16	-
HT	-	-	-	-	-	-	-	-					9
WW	-	-	-	5	-	-	40	-					-
AS	80	20	50	5	50	15	40	50	9	16	29	41	47
**Source**	(32) expert opinion	(34)	(35)	(36)	expert opinion	(20)	(36), (37), expert opinion	(10)	(11)	(12)	(13)	(14, 15)	(38)

Legend: RP – Radical Prostatectomy, RT – Radiotherapy, BY – Brachytherapy, EBRT – External Beam Radiotherapy, ADT – Androgen Deprivation Therapy, WW – Watchful Waiting, AS – Active Surveillance, HT – Hormone Therapy

Table S5. CHEERS criteria met

**Item**	**Item No**	**Recommendation**																						
**Title and abstract**			Govers et al(4)	Karlsson et al(16)	Teoh et al (17)	Kim et al (18)	Nicholson et al (19)	Boutell et al(20)	NICE guideline(21)	Cerantola et al (22)	Schiffer et al(23)	Dijkstra et al(24)	Nichol et al(25)	Heijnsdijk et al(26)	Mowatt et al(27)	Venderink et al(28)	Govers et al(29)	Faria et al(30)	Pahwa et al(31)	de Rooij et al(32)	Barnett et al(33)	Sathianathen et al(34)	Barnett et al (5)	Callender et al(35)
Title	1	Identify the study as an economic evaluation or use more specific terms such as "cost-effectiveness analysis", and describe the interventions compared.	1	1	1	1	1	1	0	1	0	1	0	0	1	1	1	1	1	1	1	1	1	1
Abstract	2	Provide a structured summary of objectives, perspective, setting, methods (including study design and inputs), results (including base case and uncertainty analyses), and conclusions.	1	1	1	1	1	1	0	1	1	1	1	1	1	1	1	1	1	1	1	1	1	1
**Introduction**																								
Background and objectives	3	Provide an explicit statement of the broader context for the study.	1	1	1	1	1	1	1	1	1	1	1	1	1	1	1	1	1	1	1	1	1	1
		Present the study question and its relevance for health policy or practice decisions.	1	1	1	1	1	1	1	1	0	1	1	1	1	1	1	1	1	1	1	1	1	1

**Methods**																								
Target population and subgroups	4	Describe characteristics of the base case population and subgroups analysed, including why they were chosen.	1	0	1	1	1	1	1	1	1	1	1	1	1	1	1	1	1	1	1	1	1	1
Setting and location	5	State relevant aspects of the system(s) in which the decision(s) need(s) to be made.	1	1	1	1	1	1	1	1	1	1	1	1	1	1	1	1	1	1	1	1	1	1
Study perspective	6	Describe the perspective of the study and relate this to the costs being evaluated.	0	1	0	0	0	1	1	1	0	1	0	0	1	1	1	1	0	1	0	1	1	1
Comparators	7	Describe the interventions or strategies being compared and state why they were chosen.	1	1	1	1	1	1	1	1	1	1	1	1	1	1	1	1	1	1	1	1	1	1
Time horizon	8	State the time horizon(s) over which costs and consequences are being evaluated and say why appropriate.	1	1	1	0	1	0	1	1	0	1	1	1	1	1	0	1	1	1	1	1	0	1
Discount rate	9	Report the choice of discount rate(s) used for costs and outcomes and say why appropriate.	1	1	1	0	1	2	1	1	0	1	1	1	1	1	1	1	1	1	1	1	1	1
Choice of health outcomes	10	Describe what outcomes were used as the measure(s) of benefit in the evaluation and their relevance	1	1	1	1	1	0	1	1	0	1	0	1	1	1	1	1	1	1	1	1	1	1

		for the type of analysis performed.																						
Measurement of effectiveness	11a	*Single study-based estimates:* Describe fully the design features of the single effectiveness study and why the single study was a sufficient source of clinical effectiveness data.	0	0	0	1	2	0	2	0	1	0	0	2	2	0	0	1	2	2	0	2	1	2
	11b	*Synthesis-based estimates*: Describe fully the methods used for identification of included studies and synthesis of clinical effectiveness data.	2	0	2	2	1	2	1	2	2	2	2	1	1	2	2	1	0	0	2	0	2	1
Measurement and valuation of preference based outcomes	12	If applicable, describe the population and methods used to elicit preferences for outcomes.	2	2	2	2	2	2	2	2	2	2	2	2	2	2	2	2	2	2	2	2	2	2
Estimating resources and costs	13a	*Single study-based economic evaluation:* Describe approaches used to estimate resource use associated with the alternative interventions. Describe primary or secondary research methods for	2	2	2	2	2	2	2	2	2	2	2	2	2	2	2	2	2	2	2	2	2	2

		valuing each resource item in terms of its unit cost. Describe any adjustments made to approximate to opportunity costs.																						
	13b	*Model-based economic evaluation:* Describe approaches and data sources used to estimate resource use associated with model health states. Describe primary or secondary research methods for valuing each resource item in terms of its unit cost. Describe any adjustments made to approximate to opportunity costs.	1	0	0	0	1	1	1	0	0	1	1	0	1	1	1	1	1	0	0	0	1	1
Currency, price date, and conversion	14	Report the dates of the estimated resource quantities and unit costs. Describe methods for adjusting estimated unit costs to the year of reported costs if necessary. Describe methods for	1	1	0	0	0	2	1	1	0	1	1	1	1	1	1	1	1	0	0	1	0	0

		converting costs into a common currency base and the exchange rate.																						
Choice of model	15	Describe and give reasons for the specific type of decision-analytical model used. Providing a figure to show model structure is strongly recommended.	1	1	1	0	1	1	1	1	1	1	0	1	1	1	1	1	1	1	0	1	1	1
Assumption s	16	Describe all structural or other assumptions underpinning the decision-analytical model.	1	1	1	0	1	0	1	1	0	1	1	0	1	1	0	1	1	0	0	0	1	1
Analytical methods	17	Describe all analytical methods supporting the evaluation. This could include methods for dealing with skewed, missing, or censored data; extrapolation methods; methods for pooling data; approaches to validate or make adjustments (such as half cycle corrections) to a model; and methods for handling population	0	1	0	0	0	0	1	0	0	0	0	0	1	0	0	0	1	0	0	0	0	1

		heterogeneity and uncertainty.																						
**Results**																								
Study parameters	18	Report the values, ranges, references, and, if used, probability distributions for all parameters. Report reasons or sources for distributions used to represent uncertainty where appropriate. Providing a table to show the input values is strongly recommended.	0	1	1	1	1	0	1	1	1	1	1	0	1	1	0	1	1	1	1	1	1	1
Incremental costs and outcomes	19	For each intervention, report mean values for the main categories of estimated costs and outcomes of interest, as well as mean differences between the comparator groups. If applicable, report incremental cost-effectiveness ratios.	0	1	1	1	1	1	1	0	0	1	1	1	1	1	1	1	1	1	0	1	0	1
Characterising uncertainty	20a	*Single study-based economic evaluation:* Describe the effects	2	2	2	0	2	2	2	2	2	2	2	2	2	2	2	2	2	2	2	2	2	2

		of sampling uncertainty for the estimated incremental cost and incremental effectiveness parameters, together with the impact of methodological assumptions (such as discount rate, study perspective).																						
	20b	*Model-based economic evaluation:* Describe the effects on the results of uncertainty for all input parameters, and uncertainty related to the structure of the model and assumptions.	0	1	1	0	1	0	1	0	0	0	0	0	1	0	0	0	1	1	0	0	0	0
Characterising heterogeneity	21	If applicable, report differences in costs, outcomes, or cost-effectiveness that can be explained by variations between subgroups of patients with different baseline characteristics or other observed variability in effects that are not reducible	1	2	2	2	2	2	2	2	2	2	2	2	1	2	2	2	2	2	2	2	2	2

		by more information.																						
**Discussion**																								
Study findings, limitations, generalisability, and current knowledge	22	Summarise key study findings and describe how they support the conclusions reached. Discuss limitations and the generalisability of the findings and how the findings fit with current knowledge.	1	1	1	1	1	1	1	1	1	1	1	1	1	1	1	1	1	1	1	1	1	1
**Other**																								
Source of funding	23	Describe how the study was funded and the role of the funder in the identification, design, conduct, and reporting of the analysis. Describe other non-monetary sources of support.	1	1	0	1	1	1	1	0	1	0	1	0	1	0	1	1	1	1	1	0	1	0
Conflicts of interest	24	Describe any potential for conflict of interest of study contributors in accordance with journal policy. In the absence of a journal policy, we recommend authors comply with International	0	1	1	1	1	1	1	0	1	1	1	1	1	0	1	1	1	1	1	1	1	1

		Committee of Medical Journal Editors recommendations.																						
Mean percentage of applicable criteria met			65	80	68	50	84	59	89	63	37	79	63	58	100	74	68	90	89	74	53	68	74	844

Table S6. ECOBIAS checklist

	Govers et al(4)	Karlsson et al(16)	Teoh et al (17)	Kim et al (18)	Nicholson et al (19)	Boutell et al(20)	NICE guideline(21)	Cerantola et al (22)	Schiffer et al(23)	Dijkstra et al(24)	Nichol et al(25)	Heijnsdijk et al(26)	Mowatt et al(27)	Venderink et al(28)	Govers et al(29)	Faria et al(30)	Pahwa et al(31)	de Rooij et al(32)	Barnett et al(33)	Sathianathen et al(34)	Barnett et al(5)	Callener et al(35)
Narrow perspective bias	No	Yes	No	No	No	No	No	No	No	No	Yes	Yes	No	No	No	No	Unclear	No	No	No	No	No
Inefficient comparator bias*	Yes	Yes	Yes	Yes	Yes	Yes	Yes	Yes	Yes	Yes	Yes	Yes	Yes	Yes	Yes	Yes	Yes	Yes	Yes	Yes	Yes	Yes
Cost measurement omission bias	Yes	Yes	Yes	Yes	Yes	Partly	Yes	Yes	No	Partly	Yes	Yes	Yes	Yes	Yes	Yes	Yes	Partly	Yes	Partly	Yes	Yes
Intermittent data collection bias	NA	NA	NA	NA	NA	NA	NA	NA	NA	NA	NA	NA	NA	Yes	NA	NA	NA	NA	NA	NA	NA	NA
Invalid valuation bias	Partly	Partly	No	Partly	Yes	No	Yes	Partly	No	No	Partly	No	Yes	Yes	No	No	Yes	No	No	Partly	Yes	Yes
Ordinal ICER bias	Yes	Yes	Yes	Yes	Yes	Yes	Yes	Yes	Yes	Yes	Yes	Yes	Yes	Yes	Yes	Yes	Yes	Yes	Yes	Yes	Yes	Yes
Double-counting bias	No	No	No	Yes	No	No	Unclear	Unclear	Unclear	Unclear	Unclear	Unclear	Unclear	Unclear	Unclear	Unclear	Unclear	Unclear	Unclear	Unclear	Unclear	Unclear

Inappropriate discounting bias	Yes	Yes	Yes	No	Yes	NA	Yes	Yes	No	Yes	Yes	Yes	Yes	Yes	Yes	Yes	Yes	Yes	Yes	Yes	Yes	Yes
Limited sensitivity analysis bias§	No	Partly	No	No	Partly	No	Partly	No	No	No	No	No	Yes	No	No	No	Partly	No	No	No	No	No
Sponsor bias	Unclear	Yes	Yes	Yes	Yes	Yes	Yes	No	Yes	No	Yes	No	Yes	No	Yes	Yes	Yes	Yes	Yes	Yes	Yes	No
Reporting and dissemination bias	Unclear	Unclear	Unclear	Unclear	Yes	Unclear	No	Unclear	Unclear	Unclear	Unclear	Unclear	Yes	Unclear	Unclear	Unclear	Unclear	Unclear	Unclear	Unclear	Unclear	Unclear
Structural assumptions bias	No	Yes	Yes	Partly	Yes	Yes	Yes	Partly	Partly	Partly	No	Yes	Yes	Partly	Partly	Partly	Partly	Partly	Yes	Partly	Yes	Partly
No treatment comparator bias*	Yes	Yes	Yes	Yes	Yes	Yes	Yes	Yes	Yes	Yes	Yes	Yes	Yes	Yes	Yes	Yes	Yes	Yes	Yes	Yes	Yes	Yes
Wrong model bias	Partly	Yes	Partly	Yes	Yes	Partly	Yes	Partly	Partly	Yes	Partly	Yes	Yes	Yes	Partly	Yes	Yes	Yes	Partly	Partly	Yes	Partly
Limited time horizon bias	Yes	Yes	Yes	No	Yes	No	Yes	Yes	No	Yes	Yes	Yes	Yes	Yes	No	Yes	Yes	No	Yes	Yes	Unclear	Yes
Bias related to data identification	Partly	Partly	No	Partly	Partly	No	Yes	Partly	Partly	Partly	Partly	Partly	Yes	Partly	No	Yes	Yes	Partly	Partly	Partly	Partly	Partly
Bias related to baseline data	NA	Yes	Unclear	Yes	Yes	Unclear	Yes	Unclear	Yes	Unclear	Unclear	Unclear	Yes	Unclear	Unclear	Yes	Unclear	Unclear	Unclear	Unclear	Unclear	Yes
Bias related to treatment effects	NA	NA	NA	NA	NA	NA	Yes	NA	NA	NA	NA	NA	Yes	NA	NA	NA	NA	NA	NA	NA	NA	NA
Bias related to quality-of-life weights (utilities)	Yes	Yes	Yes	NA	Yes	NA	Yes	Yes	NA	Yes	Yes	Yes	Yes	Yes	Yes	Yes	Yes	Yes	Yes	Yes	Yes	Yes
Non-transparent data incorporation bias	Yes	Yes	No	Partly	Yes	No	Yes	Partly	Yes	Partly	Yes	No	Yes	Partly	No	Partly	Yes	Yes	Partly	Partly	Partly	Yes

Limited scope bias§	No	Partly	No	No	Partly	No	Partly	No	No	No	No	No	Yes	No	No	No	No	No	No	No	No	No
Bias related to internal consistency	Unclear	Unclear	Unclear	Unclear	Unclear	Unclear	Unclear	Unclear	Unclear	Unclear	Unclear	Yes	Yes	Unclear	Unclear	Unclear	Unclear	Unclear	Unclear	Unclear	Unclear	Unclear

APPENDIX REFERENCES

1. Løgager VB. MRI of the Prostate: A Practical Approach. SAGE Publications Sage UK: London, England; 2018.
2. Ström P, Nordström T, Aly M, Egevad L, Grönberg H, Eklund M. The Stockholm-3 Model for Prostate Cancer Detection: Algorithm Update, Biomarker Contribution, and Reflex Test Potential. European Urology. 2018;74(2):204-10.
3. Institute NC. 18F-choline 2021 [Available from: https://www.cancer.gov/publications/dictionaries/cancer-terms/def/18f-choline.
4. Govers TM, Hessels D, Vlaeminck-Guillem V, Schmitz-Drager BJ, Stief CG, Martinez-Ballesteros C, et al. Cost-effectiveness of SelectMDx for prostate cancer in four European countries: a comparative modeling study. Prostate Cancer Prostatic Dis. 2019;22(1):101-9.
5. Barnett CL, Davenport MS, Montgomery JS, Kunju LP, Denton BT, Piert M. (18)F-Choline PET/mpMRI for Detection of Clinically Significant Prostate Cancer: Part 2. Cost-Effectiveness Analysis. J Nucl Med. 2019;60(12):1705-12.
6. Davenport MS, Montgomery JS, Kunju LP, Siddiqui J, Shankar PR, Rajendiran T, et al. ¹⁸F-Choline PET/mpMRI for Detection of Clinically Significant Prostate Cancer: Part 1. Improved Risk Stratification for MRI-Guided Transrectal Prostate Biopsies. Journal of Nuclear Medicine. 2020;61(3):337-43.
7. Haas GP, Delongchamps NB, Jones RF, Chandan V, Serio AM, Vickers AJ, et al. Needle Biopsies on Autopsy Prostates: Sensitivity of Cancer Detection Based on True Prevalence. JNCI: Journal of the National Cancer Institute. 2007;99(19):1484-9.
8. Siddiqui MM, Rais-Bahrami S, Turkbey B. Comparison of MR/ultrasound fusion-guided biopsy with ultrasound-guided biopsy for the diagnosis of prostate cancer. JAMA. 2015;313:390.
9. Kim L, Boxall N, George A, Burling K, Acher P, Aning J, et al. Clinical utility and cost modelling of the phi test to triage referrals into image-based diagnostic services for suspected prostate cancer: the PRIM (Phi to RefIne Mri) study. BMC Med. 2020;18(1):95.
10. Liu J, Womble PR, Merdan S, Miller DC, Montie JE, Denton BT. Factors Influencing Selection of Active Surveillance for Localized Prostate Cancer. Urology. 2015;86(5):901-5.

11. Miñana B, Rodríguez-Antolín A, Gómez-Veiga F, Hernández C, Suárez JF, Fernández-Gómez JM, et al. Treatment trends for clinically localised prostate cancer. National population analysis: GESCAP group. Actas Urol Esp. 2016;40(4):209-16.
12. Trama A, Botta L, Nicolai N, Rossi PG, Contiero P, Fusco M, et al. Prostate cancer changes in clinical presentation and treatments in two decades: an Italian population-based study. Eur J Cancer. 2016;67:91-8.
13. Weissbach L, Stuerzebecher S, Mumperow E, Klotz T, Schnell D. HAROW: the first comprehensive prospective observational study comparing treatment options in localized prostate cancer. World J Urol. 2016;34(5):641-7.
14. Cussenot O, Rozet F, Ruffion A, Mottet N, Bordier B, Malavaud B, et al. Prise en charge du cancer de la prostate: analyse rétrospective de 808 hommes biopsiés en France. Progrès en urologie. 2013;23(5):347-55.
15. Léon P, Cancel-Tassin G, Koutlidis N, Calves J, Funes de la Vega M, Fournier G, et al. Prévalence et diversité de la prise en charge des patients atteints de cancer de la prostate classés à faible risque selon la classification de d'Amico ou le score de CAPRA : étude française multicentrique. Progrès en Urologie. 2017;27(3):158-65.
16. Karlsson A, Jauhiainen A, Gulati R, Eklund M, Gronberg H, Etzioni R, et al. A natural history model for planning prostate cancer testing: Calibration and validation using Swedish registry data. PLoS ONE [Electronic Resource]. 2019;14(2):e0211918.
17. Teoh JYC, Chiu PKF, Yip SY, Yee CH, Li SY, Ng CF. The cost-effectiveness of prostate health index for prostate cancer detection in chinese men. Int J Urol. 2018;25:342.
18. Kim L, Boxall N, George A, Burling K, Acher P, Aning J, et al. Clinical utility and cost modelling of the phi test to triage referrals into image-based diagnostic services for suspected prostate cancer: the PRIM (Phi to RefIne Mri) study. BMC Med. 2020;18(1):95.
19. Nicholson A, Mahon J, Bol, A, Beale S, Dwan K, et al. The clinical effectiveness and cost-effectiveness of the PROGENSA prostate cancer antigen 3 assay and the Prostate Health Index in the diagnosis of prostate cancer: a systematic review and economic evaluation. Health Technology Assessment (Winchester, England). 2015;19(87):i-xxxi, 1-191.
20. Bouttell J, Teoh J, Chiu PK, Chan KS, Ng CF, Heggie R, et al. Economic evaluation of the introduction of the Prostate Health Index as a rule-out test to avoid unnecessary biopsies in men with prostate specific antigen levels of 4-10 in Hong Kong. PLoS ONE [Electronic Resource]. 2019;14(4):e0215279.
21. NICE. Prostate cancer: diagnosis and management. 2019.
22. Cerantola Y, Dragomir A, Tanguay S, Bladou F, Aprikian A, Kassouf W. Cost-effectiveness of multiparametric magnetic resonance imaging and targeted biopsy in diagnosing prostate cancer. Urologic Oncology: Seminars and Original Investigations. 2016;34(3):119.e1-.e9.
23. Schiffer E, Bick C, Grizelj B, Pietzker S, Schofer W. Urinary proteome analysis for prostate cancer diagnosis: cost-effective application in routine clinical practice in Germany. Int J Urol. 2012;19(2):118-25.
24. Dijkstra S, Govers TM, Hendriks RJ, Schalken JA, Van Criekinge W, Van Neste L, et al. Cost-effectiveness of a new urinary biomarker-based risk score compared to standard of care in prostate cancer diagnostics - a decision analytical model. BJU Int. 2017;120(5):659-65.
25. Nichol MB, Wu J, Huang J, Denham D, Frencher SK, Jacobsen SJ. Cost-effectiveness of Prostate Health Index for prostate cancer detection. 2012;110(3):353-62.
26. Heijnsdijk EAM, Denham D, de Koning HJ. The Cost-Effectiveness of Prostate Cancer Detection with the Use of Prostate Health Index. Value Health. 2016;19(2):153-7.

27. Mowatt G, Scotl, G, Boachie C, Cruickshank M, Ford JA, et al. The diagnostic accuracy and costeffectiveness of magnetic resonance spectroscopy and enhanced magnetic resonance imaging techniques in aiding the localisation of prostate abnormalities for biopsy: A systematic review and economic evaluation. Health Technol Assess. 2013;17(20):i-ix+281.
28. Venderink W, Govers TM, De Rooij M, Futterer JJ, Sedelaar JPM. Cost-effectiveness comparison of imaging-guided prostate biopsy techniques: Systematic transrectal ultrasound, direct in-bore MRI, and image fusion. American Journal of Roentgenology. 2017;208(5):1058-63.
29. Govers TM, Caba L, Resnick MJ. Cost-Effectiveness of Urinary Biomarker Panel in Prostate Cancer Risk Assessment. J Urol. 2018;200(6):1221-6.
30. Faria R, Soares MO, Spackman E, Ahmed HU, Brown LC, Kaplan R, et al. Optimising the Diagnosis of Prostate Cancer in the Era of Multiparametric Magnetic Resonance Imaging: A Cost-effectiveness Analysis Based on the Prostate MR Imaging Study (PROMIS). Eur Urol. 2018;73(1):23-30.
31. Pahwa S, Schiltz NK, Ponsky LE, Lu Z, Griswold MA, Gulani V. Cost-effectiveness of MR Imaging-guided Strategies for Detection of Prostate Cancer in Biopsy-Naive Men. Radiology. 2017;285(1):157-66.
32. de Rooij M, Crienen S, Witjes JA, Barentsz JO, Rovers MM, Grutters JP. Cost-effectiveness of magnetic resonance (MR) imaging and MR-guided targeted biopsy versus systematic transrectal ultrasound-guided biopsy in diagnosing prostate cancer: a modelling study from a health care perspective. Eur Urol. 2014;66:430.
33. Barnett CL, Davenport MS, Montgomery JS, Wei JT, Montie JE, Denton BT. Cost-effectiveness of magnetic resonance imaging and targeted fusion biopsy for early detection of prostate cancer. BJU Int. 2018;122(1):50-8.
34. Sathianathen NJ, Kuntz KM, Alarid-Escudero F, Lawrentschuk NL, Bolton DM, Murphy DG, et al. Incorporating Biomarkers into the Primary Prostate Biopsy Setting: A Cost-Effectiveness Analysis. J Urol. 2018;200(6):1215-20.
35. Callender T, Emberton M, Morris S, Pharoah PDP, Pashayan N. Benefit, Harm, and Cost-effectiveness Associated With Magnetic Resonance Imaging Before Biopsy in Age-based and Risk-stratified Screening for Prostate Cancer. JAMA Network Open. 2021;4(3):e2037657-e.
